# The association between Diet Quality Index–International score and risk of diminished ovarian reserve: a case–control study

**DOI:** 10.3389/fnut.2023.1277311

**Published:** 2023-11-29

**Authors:** Rahele Ziaei, Hatav Ghasemi-Tehrani, Minoo Movahedi, Maryam Kalatehjari, Mahdi Vajdi, Amin Mokari-Yamchi, Mahshid Elyasi, Abed Ghavami

**Affiliations:** ^1^Department of Community Nutrition, School of Nutrition and Food Science, Isfahan University of Medical Sciences, Isfahan, Iran; ^2^Fertility Department, School of Medicine, Isfahan University of Medical Sciences, Isfahan, Iran; ^3^Department of Obstetrics and Gynecology, School of Medicine, Isfahan University of Medical Sciences, Isfahan, Iran; ^4^Reproductive Sciences and Sexual Health Research Center, Isfahan University of Medical Sciences, Isfahan, Iran; ^5^Student Research Committee, Department of Community Nutrition, School of Nutrition and Food Science, Isfahan University of Medical Sciences, Isfahan, Iran; ^6^Maternal and Childhood Obesity Research Center, Urmia University of Medical Sciences, Urmia, Iran; ^7^Department of Clinical Nutrition, School of Nutrition and Food Science, Shiraz University of Medical Sciences, Shiraz, Iran

**Keywords:** Diet Quality Index-International, diminished ovarian reserve, antimullerian, antral follicle count, infertility

## Abstract

**Introduction:**

Although limited evidence exists on the beneficial reproductive effects of diet quality indices, the association is still largely unknown. We aimed to investigate the association between Diet Quality Index-International (DQI-I) and antral follicle count (AFC) and serum antimullerian hormone (AMH) as precise and sensitive markers of ovarian reserve and to assess the risk of diminished ovarian reserve (DOR) in women seeking fertility treatments.

**Methods:**

In a case-control study, 370 women (120 women with DOR and 250 women with normal ovarian reserve as controls), matched by age and body mass index (BMI), were recruited. Dietary intake was obtained using a validated 80-item semi-quantitative food frequency questionnaire (FFQ). The quality of diets was assessed using DQI-I, which included four major dietary components: variety (0–20 points), adequacy (0–40 points), moderation (0–30 points), and overall balance (0–10 points). DQI-I score was categorized by quartiles based on the distribution of controls. AFC, serum AMH and anthropometric indices were measured. Logistic regression models were used to estimate multivariable odds ratio (OR) of DOR across quartiles of DQI-I score.

**Results:**

Increased adherence to DQI-I was associated with higher AFC in women with DOR. After adjusting for potential confounders, the odds of DOR decreased with increasing DQI-I score (0.39; 95% CI: 0.18–0.86).

**Conclusion:**

Greater adherence to DQI-I, as a food and nutrient-based quality index, may decrease the risk of DOR and improve the ovarian reserve in women already diagnosed with DOR. Our findings, though, need to be verified through prospective studies and clinical trials.

## Introduction

Diminished ovarian reserve (DOR) is defined by the decreased number and quality of remaining oocytes affecting nearly 10% of women seeking fertility treatments (1). Women with DOR, while having regular menstrual cycles, exhibit reduced fecundity or response to ovarian stimulation compared to women of the same age. Antral follicle count (AFC) and serum antimullerian hormone (AMH) have emerged as most widely used measures of ovarian reserve (2). DOR has been shown to be associated with early decline in reproductive function and infertility, poor response to ovarian stimulation and ART outcome, and recurrent pregnancy loss (3, 4).

Several potential etiologies were proposed to cause DOR including genetic, autoimmune, iatrogenic, and environmental factors; however, the exact etiology of DOR remains idiopathic in most cases (5). Females of the same age have various reproductive potential, which highlights the impact of environmental factors on ovarian reserve (6). Identifying modifiable lifestyle factors, such as diet, which could promote ovarian reserve and influence human fertility has been the focus of several recent observational studies (7, 8). However, these studies mainly consider individual nutrients, food or food groups such as serum 25-hydroxyvitamin D, dietary fats, and soy products in the diet (9–12). Cumulative evidence stresses the need to consider the broader spectrum of dietary factors, such as diet quality indices, rather than single nutrient-based or single food-based approaches. Such indices are created to measure adherence to dietary guidelines or certain dietary patterns and to predict the risk of chronic diseases. Adherence to diet quality indices have been associated with anti-oxidative, anti-inflammatory, and cardiometabolic benefits (13–15). Although limited evidence exists on the beneficial reproductive effects of these dietary indices, the association is still largely unknown, especially in women (16, 17).

Diet Quality Index–International (DQI-I), as a nutrient- and food-group-based index, was first created for cross-national comparisons of diet quality and to assess the risk of chronic diseases (18). The index is based on international recommendations provided by the Food and Agricultural Organization (FAO) and the World Health Organization (WHO). There are four main categories of the index: dietary variety, adequacy, moderation, and overall balance. Evidence exists on the association between DQI-I and reduced weight gain and fat mass in children (19), decreased risk of cardiovascular diseases (20), and better weight management in adults (21). The evidence on the association between diet quality indices and female fertility is scarce. We aimed to investigate the association between DQI-I and DOR in a case–control study of women who were referred to infertility centers.

## Methods

This case–control study was performed by recruiting 370 women, including 120 women with DOR and 250 women with normal ovarian reserve as controls, aged between 18 and 45 years and with body mass index (BMI) between 20 and 35 kg/m^2^. The women were recruited from infertility centers through purposive sampling. The participants were not eligible to enter the study if they (i) had a history of ovarian surgery, chemotherapy or radiotherapy, premature ovarian failure, infertility treatment, endometriosis, endocrine disorders including polycystic ovary syndrome, thyroid disorders, diabetes or impaired glucose tolerance, Cushing's syndrome, hyperprolactinemia, and androgenic disorders, or a major chronic disease (e.g., gastrointestinal diseases, cancer, cardiovascular diseases, liver or kidney disorders and mood disorders, all based on patients' medical records); (ii) were current or previous (within the last 3 months) users of oral contraceptive drugs, hormone therapy, weight-loss interventions, and multivitamin mineral supplements; (iii) were following specific diet or physical activity programs; or (iv) were current smokers or consumed alcohol. Also, women with incomplete FFQ, who answered <35 items of the FFQ, and those with implausible total energy intake (<500 and >3,500 kcal/day) were excluded. We matched women in the case and control groups based on age variable and BMI [for three subgroups: BMI values of 18.5–24.9 (normal weight), 25–29.9 (overweight), ≥30 (obesity)]. DOR diagnosis was made by an expert gynecologist, when women had either low AMH level (≤0.7 ng/mL) or low AFC (≤4 in both ovaries); in both cases, they were considered to have decreased ovarian reserve (2). Women with normal ovarian reserve were randomly selected from the same infertility center. Written informed consent was obtained from all the participants prior to their recruitment into this research. This study was approved by the research council and the local Ethics Committee of Isfahan University of Medical Sciences (IR.ARI.MUI.REC.1401.297).

### Dietary intake and physical activity measurements

For measuring dietary intake over the previous year, a validated semi-quantitative food frequency questionnaire (FFQ), which encompassed 80 food items, was used (22). Women were asked how often, on average, over the previous year, they had consumed each food item in the questionnaire. Quantifications of food items were done using commonly used units. Six response categories per food item (never, 2–3 times/month, 1 time/week, 2–4, 5–6 times/week, and daily) were considered for each food. Data were transformed to daily intake frequency. Using standard Iranian household measures, portion sizes consumed from each food item were converted into grams (23). Daily food consumption was computed by multiplying the daily frequency of intake by portion size for each food item. After that, dietary intakes were analyzed using the Nutritionist-4 software (First Databank Inc. San Bruno, CA), modified for Iranian foods. To calculate physical activity, a short form of the International Physical Activity Questionnaire (IPAQ) was used to determine the metabolic equivalent (MET) minute per week (24). The duration and frequency of physical activity days were multiplied by the activity's MET value to get the MET minute per week (MET/min/wk). The total weekly exercise minutes were then determined by summing up the scores.

### Diet Quality Index-International

We assessed the quality of diets using the DQI-I, which included four major dietary components, including variety (0–20 points), adequacy (0–40 points), moderation (0–30 points), and overall balance (0–10 points) (18). Variety consists of two parts: the overall variety of different food groups (meat and meat products, fish and shellfish, eggs, pulses and pulse products; milk and milk products; vegetables; fruits; grains) and a variety of protein sources (meats and meat products, fishes and shellfishes, eggs, pulses and pulse products, milks and milk products). The adequacy component that includes eight elements of diet (vegetables, fruits, grains, fiber, protein, Fe, Ca, and vitamin C) must be included in the consumed food in order to provide a healthy diet. In the moderation section, scores related to the groups of total fat, saturated fat, cholesterol, sodium, and energy-boosting foods are considered. The fourth component was overall balance (proportion of each macronutrient from total energy intake and fatty acid ratio). The total DQI-I score ranged from 0 to 100, with higher scores denoting better diet quality (18).

### AFC and AMH measurements

Serum AMH levels were assessed using ELISA kit (Monobind, California, USA). Transvaginal ultrasound was performed to determine the total AFC by an infertility gynecologist, which was calculated as the sum of antral follicles measuring 2–10 mm in both ovaries on the third day of an unstimulated menstrual cycle.

### Assessment of other variables

At baseline, participants completed a general demographic questionnaire, which contained questions on age, education, occupation, obstetric history (including DOR duration, history of infertility, and previous pregnancy), anthropometric measures, history of chronic diseases, past and present use of contraceptives, dietary supplements, weight-reducing drugs or other drugs, and past and present smoking status. Body weight was measured with minimal clothing and without shoes by a digital Seca scale (Saca 831, Hamburg, Germany) to the nearest 0_·_1 kg. Height was measured in a standing position without shoes using a portable stadiometer (Seca, Hamburg, Germany) to the nearest 0.5 cm. Waist circumference (WC) and hip circumference (HC) were measured twice to the nearest 0.1 cm with a tape measure. The lowest rib and iliac crest's midpoint and the largest circumference around the buttocks were used to calculate WC and HC, respectively. The waist-to-hip ratio (WHR) was then computed by dividing the measured WC (cm) by the measured HC (cm). Body mass index (BMI) was calculated as weight divided by the square of height (kg/m^2^). Fat mass (FM) and fat free mass (FFM) were estimated using Bio-Impedance Analyzer (BIA) (Inbody 770, Inbody Co, Seoul, Korea). Both systolic and diastolic blood pressures were recorded in the sitting position and after 5 min of rest using an automated digital sphygmomanometer (Microlife Blood Pressure Monitor A100- 30, Berneck, Switzerland).

### Statistical analyses

Participants were grouped into four quartiles of DQI-I based on control values. Higher quartiles of DQI-I demonstrate higher diet quality compared to lower quartiles. The statistical analyses were carried out using SPSS (version 21.0, SPSS Inc., Chicago, Illinois, USA). One-way analysis of variance (ANOVA) and Chi-square test were used to assess the differences in continuous and categorical variables across the quartiles of the DQI-I score respectively. We used analysis of covariance (ANCOVA) to compare adjusted (for FM, BMI, Physical activity and total energy) means of AMH and AFC across the DQI-I quartiles. Potential confounding variables included in the analyses were chosen based on prior literature (8, 25) as well as Directed Acyclic Graph (DAG) (26). The multivariate logistic regression analysis was conducted to evaluate the association between quartiles of DQI-I score and the odds of DOR, as well as between one-unit increase in DQI-I score and DOR risk, after adjustment for multiple covariates in three models. Model I was adjusted for physical activity (metabolic equivalents-h/week) and energy intake (kcal/d). Model II was adjusted for confounders in Mode I plus FM (continuous) and BMI (continuous) and Model III was adjusted for confounders in Mode II plus pervious pregnancy (yes/no), socioeconomic status (low, middle, high), education level (primary, secondary or tertiary), and occupation (unemployed, self-employed, employed). A *P*-value < 0.05 was considered statistically significant.

## Results

The distribution of cases and controls according to selected sociodemographic and anthropometric variables are reported in Table 1. The mean BMI of women with DOR and controls were 29.85 and 28.75 kg/m^2^, respectively. Compared to women in the control group, women with DOR had a higher mean value of FM (38.47 vs. 36.47, *P* = 0.02). As regards anthropometric measurements, WC and WHR were significantly higher in women with DOR (102.23 vs. 91.7 and 0.9 vs. 0.86, respectively). In comparison with women in the control group, women with DOR had significantly lower serum level of AMH (0.56 vs. 4.11) and AFC count (2.34 vs. 9.59).

**Table 1:** Baseline characteristics of the study participants.

**Variable**		**Case (*N* = 120)**	**Control (*N* = 250)**	***P*-value**
Age (years)		33.37 ± 3.24	32.91 ± 3.15	0.196
BMI (kg/m^2^)		29.85 ± 2.49	28.75 ± 3.45	0.235
Weight (kg)		80.96 ± 4.78	79.26 ± 8.41	0.487
FM (kg)		38.47 ± 7.05	36.47 ± 8.91	0.020
FFM (kg)		57.99 ± 11.33	60.12 ± 11.97	0.098
WC (cm)		102.23 ± 35.95	91.70 ± 12.43	0.002
HC (cm)		109.10 ± 31.59	106.10 ± 11.57	0.316
WHR		0.90 ± 0.12	0.86 ± 0.08	0.003
SBP (mmHg)		122.18 ± 12.77	123.58 ± 14.03	0.341
DBP (mmHg)		79.41 ± 11.67	81.85 ± 10.48	0.056
Physical activity (MET/h/day)		19.05 ± 4.12	18.98 ± 4.51	0.896
Socioeconomic status (SES) (%)	Low	10 (8.3)	19 (7.6)	0.252
	Middle	50 (41.7)	127 (50.8)	
	High	60 (50)	104 (41.6)	
Education (%)	Illiterate	14 (11.7)	34 (13.6)	< 0.001
	≤ High school/diploma	31 (25.8)	121 (48.4)	
	≥College degree	75 (62.5)	95 (38)	
Occupation (%)	Housewife	82 (68.3)	184 (73.6)	< 0.001
	Employed	26 (21.7)	10 (4)	
	Student	12 (10)	56 (22.4)	
Pervious pregnancy	Yes	99 (82.5)	203 (81.2)	0.441
	No	21 (17.5)	47 (18.8)	
AFC count		2.34 ± 1.19	9.59 ± 2.24	< 0.001
AMH (ng/ml)		0.56 ± 0.71	4.11 ± 1.18	< 0.001

The general characteristics of participants across the quartiles of the DQI-I score are presented in Table 2. As shown, women with DOR had significantly higher AFC (*P* = 0.037) and lower DBP (*P* = 0.041) with higher DQI-I scores. The results of ANCOVA analysis showed differences in AFC values across the DQI-I quartiles in women with DOR (*P* = 0.045).

**Table 2:** Characteristics of study participants according to quartiles of Diet Quality Index–International (DQI-I).

**Variable**		**Case (120)**					**Control (250)**				
		**Q1**	**Q2**	**Q3**	**Q4**	*P* ^*^	**Q1**	**Q2**	**Q3**	**Q4**	*P* ^*^
Age (years)		32.73 ± 2.70	33.51 ± 2.99	33.29 ± 4.23	33.75 ± 3.00	0.658	32.69 ± 3.01	32.98 ± 3.30	33.35 ± 3.10	32.54 ± 3.18	0.504
BMI (kg/m^2^)		29.16 ± 2.36	30.46 ± 2.77	29.7 ± 2.99	29.94 ± 2.50	0.299	27.59 ± 3.85	27.71 ± 3.30	27.77 ± 3.62	27.98 ± 2.89	0.123
Weight (kg)		82.34 ± 3.94	83.18 ± 4.18	81.37 ± 3.99	82.42 ± 40.20	0.448	78.05 ± 5.11	78.34 ± 5.12	78.56 ± 4.15	77.5 ± 5.28	0.428
FM (kg)		39.33 ± 6.57	39.07 ± 9.51	39.01 ± 5.83	37.14 ± 6.17	0.542	35.95 ± 8.29	37.00 ± 9.48	36.31 ± 9.98	36.68 ± 7.61	0.172
FM (kg)		56.84 ± 11.19	59.63 ± 11.93	58.42 ± 11.03	57.34 ± 11.49	0.805	61.44 ± 13.12	59.79 ± 12.09	61.81 ± 11.77	56.63 ± 9.83	0.093
WC (cm)		111.76 ± 41.23	107.44 ± 36.65	99.03 ± 35.45	94.67 ± 31.22	0.225	91.60 ± 12.94	91.73 ± 12.29	91.84 ± 12.32	91.62 ± 12.44	1.00
HC (cm)		114.69 ± 36.65	107.27 ± 27.25	106.61 ± 26.14	108.37 ± 34.73	0.783	105.72 ± 10.64	104.61 ± 11.62	107.20 ± 11.01	107.2613.38	0.518
WHR		0.90 ± 0.10	0.92 ± 0.11	0.88 ± 0.15	0.90 ± 0.12	0.621	0.86 ± 0.07	0.87 ± 0.08	0.85 ± 0.008	0.85 ± 0.07	0.415
SBP (mmHg)		126.42 ± 12.65	122.77 ± 13.33	120.40 ± 12.30	120.22 ± 12.53	0.224	122.42 ± 13.75	125.29 ± 14.63	124.68 ± 14.11	121.40 ± 13.47	0.380
DBP (mmHg)		78.73 ± 10.38	84.92 ± 13.77	76.70 ± 10.26	77.97 ± 11.02	0.041	83.07 ± 10.91	82.27 ± 10.80	81.95 ± 10.10	79.50 ± 11.66	0.346
AFC count		2.15 ± 1.08	2.22 ± 1.15	2.15 ± 1.29	2.92 ± 1.07	0.037	9.58 ± 2.35	9.82 ± 2.17	9.57 ± 2.37	9.30 ± 2.01	0.667
						0.045^a^					0.532^a^
AMH (ng/ml)		0.47 ± 0.25	0.74 ± 1.46	0.52 ± 0.17	0.52 ± 0.19	0.496	4.01 ± 1.30	4.03 ± 1.17	4.13 ± 1.06	4.31 ± 1.18	0.548
						0.323^a^					0.445^a^
Physical activity (MET/h/day)		18.88 ± 4.04	18.85 ± 4.23	18.70 ± 4.66	19.52 ± 3.80	0.847	19.44 ± 4.59	18.38 ± 4.15	19.00 ± 4.80	19.18 ± 4.53	0.575
Socioeconomic status (SES) (%)	Low	3 (11.5)	4 (14.8)	2 (7.4)	1 (2.5)	0.435	7 (10.3)	5 (7.4)	4 (6.3)	3 (6)	0.850
	Middle	8 (30.8)	10 (37)	11 (40.7)	21 (52.5)		32 (47.1)	38 (55.9)	30 (46.9)	27 (54)	
	High	15 (57.7)	13 (48.1)	14 (51.9)	18 (45)		29 (42.6)	25 (36.8)	30 (46.9)	20 (40)	
Education (%)	Illiterate	6 (23.1)	3 (11.1)	2 (7.4)	3 (7.5)	0.199	4 (5.9)	12 (17.6)	8 (12.5)	10 (20)	0.211
	≤ High school/diploma	9 (34.6)	4 (14.8)	8 (29.6)	10 (25)		40 (58.8)	30 (44.1)	28 (43.8)	23 (46)	
	≥College degree	11 (42.3)	20 (74.1)	17 (63)	27 (67.5)		24 (35.3)	26 (38.2)	28 (43.8)	17 (34)	
Occupation	Housewife	20 (76.9)	20 (74.1)	16 (59.3)	26 (65)	0.504	51 (75)	47 (69.1)	48 (75.0)	38 (76)	0.804
	Employed	5 (19.2)	4 (14.8)	6 (22.2)	11 (27.5)		2 (2.9)	5 (7.4)	2 (3.1)	1 (2)	
	Student	1 (23.8)	‘3 (11.1)	5 (18.5)	3 (7.5)		15 (22.1)	16 (23.5)	14 (21.9)	11 (22)	
Pervious pregnancy	Yes	18 (69.2)	25 (92.6)	22 (81.5)	34 (85)	0.153	50 (73.5)	53 (77.9)	56 (87.5)	44 (88)	0.099
	No	8 (30.8)	2 (7.4)	5 (18.5)	6 (15)		18 (26.5)	15 (22.1)	8 (12.5)	6 (12)	

ANOVA test used for continuous variables; Chi-square test used for categorical variables. Quantitative variables are expressed as mean ± SD and qualitative variables expressed as n (%). The SES scored was evaluated based on education level of both subjects and the family head, job of both subjects and the family head family size, home status and home type by using self-reported questionnaire.

* P-values resulted from ANOVA test for quantitative and Chi-square for qualitative variables across quartiles.

a P ANCOVA test adjusted for FM, BMI, physical activity, and total energy. AFC, antral follicle count; BMI, body mass index; DBP, diastolic blood pressure; DOR, diminished or decreased ovarian reserve; FFM, fat free mass; FM, fat mass; HC, hip circumference; SBP, systolic blood pressure; WC, waist circumference; WHR, waist-to-hip ratio.

Odds ratio (OR) and 95% confidence intervals (CIs) for DOR across the quartiles of DQI-I score are indicated in Table 3. Participants in the top quartile of DQI-I were less likely to have DOR than those in the bottom quartile in the crude model (OR: 0.47; 95%CI: 0.25–0.88). After adjusting for potential confounders in models I, II, and III, the odds of DOR decreased with an increase in the DQI-I score (OR: 0.37; 95% CI: 0.19–0.74, 0.39; 95% CI: 0.15–0.79 and 0.37; 95% CI: 0.23–0.89, respectively). Each unit increase of DQI-I score correlated with a lower risk of DOR (OR: 0.94; 95% CI: 0.73–0.98; *P* = 0.032) in fully adjusted model (Table 3).

**Table 3:** Odds ratio (95% CI) of DOR according to quartiles of DQI-I.

	**DQI-I**						
	**Q1**	**Q2**	**Q3**	**Q4**	**P-trend**	**A one-unit increase in DQI-I score (continuous)**	
DOR/control	(26/68)	(27/68)	(27/64)	(40/50)	*P*
Crude	Ref (1.00)	0.96 (0.51–1.81)	0.90 (0.47–1.71)	0.47 (0.25–0.88)	0.019	0.79 (0.69–0.88)	0.003
Model 1	Ref (1.00)	0.73 (0.36–1.47)	0.73 (0.36–1.49)	0.37 (0.19–0.74)	0.007	0.83 (0.71–0.98)	0.008
Model 2	Ref (1.00)	0.83 (0.3–1.85)	0.67 (0.29.-1.41)	0.39 (0.15–0.79)	0.015	0.88 (0.78–0.95)	0.015
Model 3	Ref (1.00)	0.84 (0.39–1.85)	0.67 (0.33–1.44)	0.37 (0.23–0.89)	0.022	0.94 (0.73–0.98)	0.032

Model 1, Physical activity (metabolic equivalents-h/week) and energy intake (kcal/day). Model 2, model 1 + FM and BMI. Model 3, model 2 + previous pregnancy (yes/no) and socioeconomic status (low, middle, high), education level (primary, secondary, or tertiary), and occupation (unemployed, self-employed, employed). DQI-I, Diet Quality Index–International; DOR, diminished ovarian reserve.

## Discussion

To our knowledge, the present study is the first to explore the association between DQI-I score and serum AMH levels and AFC in women with or without DOR, as well as odds of decreased ovarian reserve across quartiles of DQI-I scores. Greater adherence to DQI-I was associated with higher AFC in women with DOR. Also, odds of DOR decreased with an increase in the DQI-I score.

DQI-I, as a food group and nutrient-based index, might be more advantageous for evaluating the overall quality of the diet compared to other diet quality indices, which are nutrient-based or food-based (14). As there are no previous studies examining the association between DQI-I and ovarian reserve (to the best of our knowledge), it makes the interpretation of our findings difficult. Evidence exists on the association between DQI-I and cardiometabolic health biomarkers. Adherence to DQI-I was associated with several metabolic parameters including BMI, WC, total cholesterol, Apo A/B, blood pressure, and uric acid, and DQI-I was confirmed as a good predictor of serum metabolic parameters in 1,404 adults from the Observation of Cardiovascular Risk Factors in Luxembourg (ORISCAV-LUX-2) study (14). Also, DQI-I was inversely correlated with BMI and serum total cholesterol and positively with high-density lipoprotein cholesterol (HDL-c) in other studies (20, 27).

DOR leads to early decline in reproductive function and infertility, poor response to ovarian stimulation and ART outcome, and recurrent pregnancy loss (4, 28). Unknown etiology and limited therapeutic approaches to DOR have made it as a major challenge in infertility treatment. It has been proposed that nutritional factors such as serum 25-hydroxyvitamin D level and intake of soy products might affect ovarian reserve (6). In late premenopausal women, serum AMH concentration was inversely associated with dietary fat intake (7). In a prospective study on the association between dietary intake and rate of AMH decline among eumenorrheic women from the Tehran Lipid and Glucose study, it was shown that the consumption of dairy food reduced the rate of AMH decline in these women (29). Eskew et al. (8) conducted a cross-sectional study on 185 overweight and obese women without a history of infertility from the Lifestyle and Ovarian Reserve (LORe) cohort and found that greater adherence to a profertility diet, characterized by increased intake of whole grains, soy, seafood, dairy, low pesticide residue fruit and vegetables, and supplemental vitamin D, folic acid and B12, was associated with higher AMH level and AFC. Also, low preconception Mediterranean diet score (MDS) was reported to be a risk factor for poor ovarian response in a study on 296 women seeking infertility treatments (30). Conversely, in the Environment and Reproductive Health (EARTH) Study, no significant relation between dietary patterns, including Mediterranean diet, fertility diet and profertility diet, and AFC was shown among women attending a fertility center (25).

Although the underlying mechanism of DOR remains largely unknown, oxidative stress and chronic inflammation followed by metabolic derangements have been proposed to negatively affect ovarian reserve. In this regard, antioxidant compounds were successfully used to improve ovarian reserve (31–33). Also, in a metabonomic study on the follicular fluid of women with DOR, these women had unique metabolic characteristics in their follicular fluid (28). Increased adiposity may further worsen their condition by exacerbating low and moderate inflammation and oxidative damage, as overweight and obese women with DOR have lower AMH levels compared to nonobese women with DOR (34). The association between DQI-I score and ovarian reserve may be mediated by inflammation or adiposity, as dietary quality indices were found to be inversely correlated with inflammatory markers and BMI in previous studies (14, 27, 35). In this regard, higher adherence to DQI-I and its components had a negative association with inflammatory markers including high-sensitivity C-reactive protein (hs–CRP) in a cross-sectional study on 200 Iranian overweight and obese women (36). In another study by Koohdani et al. (37) on patients with type-2 diabetes, higher scores for DQI-I were inversely associated with markers of oxidative stress.

We found no significant differences in BMI, WHR, and WC across the quartiles of DQI-I score in both case and control groups, which is consistent with a number of studies that examined the association between DQI-I and obesity measures (38, 39). Overall, studies evaluating diet quality indices and obesity revealed conflicting results, which can be attributed to their different target populations (19, 40). Most dietary quality indices were designed for the U.S. population, so they may not reflect the overall diet quality in other populations accurately, especially those from developing countries. Also, overweight and obese individuals may follow a healthier lifestyle, including diet, to manage their weight. So, the effect of a high-quality diet on their weight status cannot be predicted with accuracy.

Several strengths of this study should be noted, including its novel findings. We evaluated the diet quality of participants rather than measuring dietary intakes or serum levels of nutrients so as to consider the broader spectrum of dietary factors and to better counsel women about the nutritional factors and reproductive health. A large sample size and matching case and controls by age and BMI to reduce the effect of confounding variables were other strengths of this study. However, several limitations must be considered when interpreting our findings. The case–control design of the study limits our ability to conclude a causal association between DQI-I and the risk of DOR. Additionally, some confounders such as mood status and genetic background may not have been taken into account. Finally, despite using a validated FFQ to estimate dietary intakes, measurement error and recall bias should be considered.

## Conclusions

In this case–control study, increased adherence to DQI-I, as a food group and nutrient-based index, was associated with higher AFC in women with decreased ovarian reserve, suggesting improved ovarian reserve in these women. Also, we found that greater adherence to DQI-I may decrease the risk of DOR. Our findings suggest the possibility of reducing the risk of DOR or improving the ovarian reserve in these women by nutrition counseling regarding the importance of adopting a healthy diet or adherence to dietary guidelines. However, this finding needs to be verified through prospective studies and clinical trials.

## Data availability statement

The original contributions presented in the study are included in the article/supplementary material, further inquiries can be directed to the corresponding author.

## Ethics statement

The studies involving humans were approved by Local Ethics Committee of Isfahan University of Medical Sciences (IR.ARI.MUI.REC.1401.297). The studies were conducted in accordance with the local legislation and institutional requirements. The participants provided their written informed consent to participate in this study.

## Author contributions

RZ: Conceptualization, Investigation, Methodology, Writing – original draft, Writing – review & editing. HG-T: Conceptualization, Investigation, Writing – original draft. MM: Investigation, Writing – original draft. MK: Data curation, Methodology, Software, Writing – original draft. MV: Data curation, Formal analysis, Software, Writing – original draft. AM-Y: Data curation, Formal analysis, Software, Writing – original draft. ME: Investigation, Writing – original draft. AG: Conceptualization, Data curation, Formal analysis, Software, Writing – review & editing.
